# Effects of Crayfish Shell Meal Processing Methods on Growth Performance, Antioxidant Capacity, Intestinal Function and Gut Microbiota of Largemouth Bass (*Micropterus salmoides*)

**DOI:** 10.3390/ani16111658

**Published:** 2026-05-29

**Authors:** Zeping Fang, Chao Song, Shengyun Kang, Guoliang Ruan, Xiaofeng Huang

**Affiliations:** 1 College of Animal Science and Technology, Yangtze University, Jingzhou 434024, China; 2 Freshwater Fisheries Research Center, Chinese Academy of Fishery Sciences, Wuxi 214081, China; 3 Jiangxi Agricultural Technology Promotion Center, Nanchang 330046, China; 4 Hubei Fisheries Industrial Technology Research Institute, Jingzhou 434026, China

**Keywords:** fermentation, enzymatic hydrolysis, crustacean processing by-products, oxidative stress, intestinal inflammation, high-value utilization

## Abstract

This study evaluated the effects of crayfish shell meal (CSM) processed by drying, enzymatic hydrolysis, or fermentation on juvenile largemouth bass (*Micropterus salmoides*). A total of 320 fish (initial body weight, approximately 5.69 g) were distributed into 16 tanks, with 20 fish per tank and four replicates per treatment. Four isonitrogenous and isoenergetic diets were prepared: a fish meal-based control diet (FM) and three diets containing 4.5% dried CSM (DCSM), enzymatically hydrolyzed CSM (ECSM), or fermented CSM (FCSM), in which fish meal was reduced from 45.0% to 39.6%, and were fed for 8 weeks. Compared with the FM group, DCSM significantly reduced final body weight, weight gain rate, specific growth rate, and protein efficiency ratio, and increased feed conversion ratio (*p* < 0.05). ECSM generally showed intermediate responses, whereas FCSM did not differ significantly from FM in these growth and feed utilization indices (*p* > 0.05). DCSM also significantly increased serum aspartate aminotransferase and blood urea nitrogen levels and induced a pro-inflammatory intestinal response, as indicated by decreased *il10* and increased *il1b* and *tnfa* expression (*p* < 0.05). Compared with DCSM, FCSM significantly improved these serum and inflammatory indices, increased intestinal trypsin, Na^+^/K^+^-ATPase and γ-glutamyl transferase activities (*p* < 0.05), and showed more favorable antioxidant and gut microbiota responses. Overall, fermentation was more effective than enzymatic hydrolysis in improving the feeding value of CSM for largemouth bass. These results suggest that FCSM is a promising alternative ingredient for aquafeeds and may contribute to the high-value utilization of crayfish processing by-products.

## Simple Summary

Crayfish processing produces large amounts of shell by-products that could be used in aquafeeds, but their direct use is limited by poor digestibility and high chitin and ash contents. This study compared dried, enzymatically hydrolyzed, and fermented crayfish shell meals as partial replacements for fish meal in diets for juvenile largemouth bass. Dried crayfish shell meal reduced growth and caused less favorable intestinal and physiological responses. In contrast, fermented crayfish shell meal maintained growth performance comparable to the fish meal control and improved antioxidant status, intestinal enzyme activity, and gut microbiota. Enzymatic hydrolysis showed partial benefits but was less effective than fermentation. These results suggest that fermentation is a useful strategy for improving the feeding value of crayfish shell by-products and converting them into sustainable aquafeed ingredients.

## 1. Introduction

Fish meal has long served as a major protein source in aquafeeds, especially for carnivorous fish. However, the increasing cost and limited availability of fish meal have intensified the need to develop sustainable and nutritionally suitable alternative protein sources [1,2]. In this context, aquatic processing by-products, such as fish frames, heads, viscera, skin and trimming residues, shrimp and crab shells, cephalothorax residues, and mollusk shells, have received increasing attention because they are widely available and may help reduce feed cost and waste discharge [3,4,5]. These by-products have been successfully processed into fish meal replacers, protein hydrolysates, shell meals, silages, fermented feed ingredients, and functional feed additives in aquafeeds [4,5,6].

Red swamp crayfish (*Procambarus clarkii*) is an important aquaculture species in China, with a production of 3.45 million tons in 2024, ranking fourth among freshwater aquaculture species in China. In the same year, the processing volume of red swamp crayfish reached 1.38 million tons, indicating the generation of considerable quantities of processing by-products [7]. Its processing generates abundant by-products, mainly including cephalothorax, appendages, and shell residues. These by-products have potential value as feed ingredients because they contain approximately 34–39% crude protein, 10–11% crude lipid, 29–39% ash, and 20–30% chitin on a dry matter basis [8,9,10,11,12]. They also contain functional compounds such as astaxanthin, with reported levels of approximately 70–90 μg/g on a wet-weight basis [13,14]. However, the direct use of crayfish by-products in aquafeeds is often constrained by low digestibility, high ash content, and the compact chitin–protein–mineral matrix [5,15,16]. Moreover, when the cephalothorax is included, residual hepatopancreatic tissue may increase lipid content and make the raw material more susceptible to oxidation during processing and storage, which may further impair ingredient quality and affect fish health [17,18].

Processing treatment is therefore important before these by-products are used in feed. Drying is simple and practical, but it mainly serves as a preservation step and does not substantially improve nutrient availability. For materials with relatively high lipid content, heat treatment may also increase the risk of oxidation [18]. Enzymatic hydrolysis is considered a useful way to improve the utilization of by-products because it can break down proteins into smaller peptides and soluble components [19,20]. Fermentation is another commonly used approach. Through microbial action, fermentation can modify substrate characteristics and generate organic acids, bioactive peptides, antioxidant compounds, and extracellular enzymes such as protease and chitinase, thereby improving nutrient availability and contributing to the nutritional or functional value of the ingredient [21,22,23]. Studies have shown that microorganisms such as *Bacillus subtilis*, *Lactobacillus plantarum*, and *Saccharomyces cerevisiae* can be used in feed fermentation and may help improve antioxidant status, immune response, and intestinal health in aquatic animals [24,25].

Largemouth bass (*Micropterus salmoides*) is an important freshwater carnivorous species with a high dietary protein requirement, with reported optimal protein levels of approximately 47.8–51.6% [26,27]. Although many studies have evaluated alternative proteins for this species, the use of crayfish by-products, particularly after different processing treatments, has not been sufficiently studied [28]. In addition to growth performance, more attention should be paid to health-related responses, including antioxidant capacity and intestinal homeostasis, because these indices are closely related to the physiological condition of fish under culture conditions [29,30].

Previous studies have reported that enzymatic treatment or fermentation of animal by-products can improve their practical utilization and, in some cases, health-related value in fish [31,32,33,34]. For instance, enzymatic hydrolysis increases the contents of acid-soluble protein and small peptides in black soldier fly (*Hermetia illucens*) pulp by approximately 95.5% and 19.9%, respectively [31]. Replacing 37.5–50% of fish meal with fermented fish-offal meal improves the apparent protein digestibility, protein efficiency ratio and apparent net protein utilization of Indian minor carp (*Labeo bata*) by 2.6–5.8%, 26.7–69.3% and 42.3–58.5%, respectively [34]. Nevertheless, direct comparisons among dried, enzymatically hydrolyzed, and fermented crayfish shell meals prepared from the same raw material are still limited. Their relative effects on growth performance, oxidative balance, and intestinal health in largemouth bass remain unclear.

Although enzymatic hydrolysis and fermentation have been used to improve animal by-products in aquafeeds, their effects may vary with raw material characteristics, processing conditions, and target species. For crayfish processing by-products, the chitin–protein–mineral matrix, high ash content, and oxidation-prone lipid fraction may respond differently to drying, enzymatic hydrolysis, and fermentation. Therefore, we hypothesized that different processing methods would produce distinct effects on the feeding value of crayfish shell meal in largemouth bass. In the present study, dried crayfish shell meal (DCSM), enzymatically hydrolyzed crayfish shell meal (ECSM), and fermented crayfish shell meal (FCSM) were prepared from crayfish by-products consisting of the cephalothorax and appendages after removal of the abdomen. Juvenile largemouth bass were fed the experimental diets for 8 weeks, and the effects of these three processed products on growth performance, antioxidant capacity, intestinal homeostasis and gut microbiota were comparatively evaluated. The aim of this study was to provide a basis for the effective utilization of crayfish processing by-products in aquafeeds.

## 2. Materials and Methods

### 2.1. Treatment of CSM

Live red swamp crayfish weighing approximately 20–40 g were purchased from local markets in Jingzhou City, China. The crayfish were sourced from local integrated rice-crayfish farming bases and transported to the laboratory on the same day. After rinsing and draining, the abdomens were manually removed, whereas the cephalothoraxes and appendages were retained as crayfish processing by-products. The retained by-product material was thoroughly homogenized and divided into three portions for the preparation of dried meal, enzymatically hydrolyzed meal, and fermented meal. For dried meal preparation, fresh crayfish by-products were dried in a forced-air oven at 65 °C until constant weight was reached. The dried material was then subjected to heat treatment at 105 °C for 15 min to reduce microbial contamination. For enzymatically hydrolyzed meal preparation, fresh crayfish by-products, neutral protease preparation (50 U/mg; Macklin, Shanghai, China), and deionized water were mixed at a ratio of 10:1:5 (*w*/*w*/*w*). The mixture was incubated at 50 °C for 7 h with continuous stirring. After hydrolysis, the enzyme was inactivated by heating. The product was subsequently dried and stored at −20 °C until diet preparation. For fermented meal preparation, *Bacillus subtilis* CGMCC 1.933, *Lactobacillus plantarum* CGMCC 1.557, and *Saccharomyces cerevisiae* CGMCC 2.1416 were purchased from the China General Microbiological Culture Collection Center (CGMCC, Beijing, China). Each strain was activated separately and adjusted to approximately 1.0 × 10^8^ CFU/mL prior to inoculation. The three cultures were mixed at a ratio of 1:1:1 (*v*/*v*/*v*) to prepare a compound inoculum. The initial pH of the fresh crayfish by-product substrate was not adjusted before inoculation. Fresh crayfish by-products were inoculated with 7.5–10% (*v*/*w*) inoculum, and the moisture content was adjusted to 50–55%. Brown sugar was added at 0.5% as an auxiliary carbon source. Fermentation was conducted sequentially under aerobic conditions at 30–32 °C for 24 h, under anaerobic conditions in sealed bags at 28–30 °C for 48 h, and then under sealed post-fermentation conditions at 25–28 °C for another 48 h. The pH values before and after fermentation were determined by mixing 10 g of the sample with 90 mL of distilled water and measuring the supernatant using a calibrated pH meter. The initial pH was 6.6 ± 0.1, whereas the final pH decreased to 4.8 ± 0.2 after fermentation. The material was then air-dried at room temperature to a moisture content below 10% and stored at −20 °C.

### 2.2. Experimental Diets

Four isonitrogenous and isoenergetic diets were formulated for the feeding trial. The control diet (FM) contained 45.0% fish meal, whereas the DCSM, ECSM, and FCSM diets contained 39.6% fish meal and 4.5% dried, enzymatically hydrolyzed, or fermented crayfish shell meal, respectively. Because this study aimed to compare processing methods rather than determine the optimal CSM inclusion level, a moderate inclusion level was selected to reduce confounding effects from the high ash, calcium, and chitin contents of CSM. The other ingredients were adjusted accordingly to maintain comparable crude protein and gross energy levels among diets and to avoid excessive Ca:P imbalance.

The ingredient formulation and the chemical composition of the experimental diets are shown in Table 1. All dry ingredients were ground, passed through a 60-mesh sieve, and thoroughly mixed. After the addition of oils and water, the mash was pelleted, dried, and stored at −20 °C until use.

### 2.3. Fish and Feeding Trial

Juvenile largemouth bass were purchased from a commercial fish farm in Hubei Province, China, and then transferred to the Aquatic Animal Breeding Base of Yangtze University for temporary rearing and feeding trials. The temporary rearing period lasted 2 weeks, during which the experimental fish were fed the basal diet of the FM group. Subsequently, 320 healthy fish with similar body weights (initial body weight, 5.69 ± 0.18 g; mean ± SD) were selected, weighed individually, and randomly allocated to 16 tanks (water volume 240 L), with 4 replicates per dietary treatment. The feeding trial lasted 8 weeks and was performed in an indoor static-water culture system. Fish were fed to apparent satiation twice daily at 08:00 and 18:00 by the same trained personnel throughout the feeding trial. Feed was supplied slowly, and feeding was stopped when fish showed no active feeding response for approximately 5 min. The same feeding procedure and observation criteria were applied to all tanks and treatments. Municipal tap water was used as culture water, which was stored in large water-storage barrels and continuously aerated for dechlorination prior to use. Each rearing tank was equipped with an air stone connected to an air pump for continuous aeration. Feces were siphoned before each feeding, and uneaten feed was collected by siphoning approximately 1 h after feeding and stored at −20 °C. During daily maintenance, one-third to two-thirds of the tank water was renewed to maintain favorable water quality. The room temperature was regulated by air-conditioning units to stabilize the water temperature. During the feeding trial, water temperature was maintained at 26 ± 2 °C, pH 7.0–7.5, dissolved oxygen ≥ 6.0 mg/L, and ammonia nitrogen < 0.5 mg/L. At the end of the experiment, the uneaten feed was dried and weighed to calculate the actual feed intake. All animal experimental procedures were conducted in accordance with the guidelines for the care and use of laboratory animals and were approved by the Animal Ethics Committee of Yangtze University.

### 2.4. Sample Collection

After the completion of the feeding trial, the experimental fish were fasted for 24 h to empty their intestinal contents. The fish were anesthetized with MS-222 (Aladdin, Shanghai, China) at 100 mg/L before sampling, and all fish in each tank were counted and weighed to calculate growth performance and survival rate.

For the determination of whole-body proximate chemical composition, 3 fish were randomly selected from each tank. Another 6 fish were randomly chosen from each tank, weighed individually, and dissected. The visceral mass and liver were separated and weighed to calculate the viscerosomatic index (VSI) and hepatosomatic index (HSI). The stomach and intestinal tissues were then carefully dissected, rinsed with ice-cold physiological saline together with the previously collected liver samples, immediately snap-frozen in liquid nitrogen, and stored at −80 °C for subsequent analysis. Among these samples, liver and intestinal tissues were used for the determination of antioxidant capacity, while stomach and intestinal tissues were used for the analysis of digestive enzyme and intestinal-related enzyme activities. Dorsal muscle samples were also collected for proximate chemical composition analysis of muscle.

Blood samples were collected from the caudal vein of the remaining fish using a sterile syringe, allowed to clot at 4 °C for 4 h, and then centrifuged at 1200× *g* for 10 min at 4 °C to obtain serum [35]. Serum samples were stored at −80 °C until analysis. Then, 3 fish were randomly selected from each tank for gut microbiota sampling. The midgut and hindgut contents from the 3 fish within the same tank were aseptically collected and pooled into one sterile cryogenic vial, which was considered one biological replicate. The pooled samples were immediately snap-frozen in liquid nitrogen and stored at −80 °C until gut microbiota analysis. Additionally, midgut tissues were collected from 3 fish per tank for total RNA extraction.

### 2.5. Calculation of Growth Performance and Somatic Indices

Growth performance indices and somatic indices, including weight gain rate (WGR), specific growth rate (SGR), feed conversion ratio (FCR), protein efficiency ratio (PER), feed intake (FI), feeding rate (FR), survival rate (SR), hepatosomatic index (HSI), and viscerosomatic index (VSI), were calculated according to the following equations:

(1) $$WGR\;\left(\%\right)=\left[\left(FBW-IBW\right)÷IBW\right]\times 100$$

(2) $$SGR\;(\%/d)=\left[\left(\ln FBW-\ln IBW\right)÷t\right]\times 100$$

(3) $$FCR=total\;feed\;intake÷\left(FBW-IBW\right)$$

(4) $$PER=\left(FBW-IBW\right)÷total\;protein\;intake$$

(5) $$FR\;(\%BW/d)=total\;feed\;intake÷\left\{t\times \left[\left(IBW+FBW\right)÷2\right]\right\}\times 100$$

(6) $$FI\;(g/fish/d)=total\;feed\;intake÷\left(final\;fish\;number\times t\right)$$

(7) $$SR\;(\%)=\left(final\;fish\;number÷initial\;fish\;number\right)\times 100$$

(8) $$HSI\;(\%)=\left(liver\;weight÷body\;weight\right)\times 100$$

(9) $$VSI\;(\%)=\left(viscera\;weight÷body\;weight\right)\times 100$$

where FBW is final body weight, IBW is initial body weight, and *t* is the feeding trial duration in days.

### 2.6. Proximate Composition Analysis

The proximate composition of the experimental diets, whole body, and dorsal muscle was determined according to standard AOAC procedures [36]. Moisture content was measured by drying samples at 105 °C to constant weight. Crude protein content and crude lipid content were determined by the Kjeldahl method and the Soxhlet extraction method. Ash content was determined by incineration in a muffle furnace at 550 °C for 6 h. Calcium and phosphorus contents in the diets were determined according to the Chinese National Standards GB/T 6436-2018 [37] and GB/T 6437-2018 [38], respectively.

### 2.7. Serum Biochemical, Antioxidant Capacity, and Digestive and Intestinal Enzyme Activity Analyses

Unless otherwise stated, all biochemical parameters were determined using commercial assay kits purchased from Nanjing Jiancheng Bioengineering Institute (Nanjing, China) following the manufacturer’s instructions. Serum samples were directly used for the determination of biochemical parameters, including total protein (TP), albumin (ALB), alanine aminotransferase (ALT), aspartate aminotransferase (AST), triglycerides (TG), total cholesterol (TC), glucose (GLU), and blood urea nitrogen (BUN).

For tissue sample preparation, liver, stomach, and intestinal tissues were homogenized in 9 volumes of ice-cold physiological saline at a mass-to-volume ratio of 1:9, then centrifuged at 850× *g* for 10 min at 4 °C. The supernatants were collected for subsequent analysis. The protein concentration in tissue homogenates was measured using a commercial protein assay kit, and all tissue antioxidant indices and enzyme activity data were normalized to protein concentration. Antioxidant capacity in serum, liver, and intestine was evaluated by measuring the activities of superoxide dismutase (SOD), catalase (CAT), glutathione peroxidase (GSH-Px), as well as total antioxidant capacity (T-AOC) and malondialdehyde (MDA) content. Digestive enzyme indices included pepsin, lipase, and amylase in the stomach, as well as trypsin, lipase, and amylase in the intestine. Intestinal functional enzyme activities were also determined, including Na^+^/K^+^-ATPase, alkaline phosphatase (AKP), γ-glutamyl transferase (γ-GT), and creatine kinase (CK).

### 2.8. Total RNA Extraction and Gene Expression Analysis

Total RNA was extracted from midgut tissues using RNAiso Plus reagent (Takara, Dalian, China). RNA concentration and purity were assessed spectrophotometrically, and RNA integrity was verified by agarose gel electrophoresis. First-strand cDNA was synthesized using the PrimeScript^TM^ RT reagent Kit with gDNA Eraser (Takara, Dalian, China). Quantitative real-time PCR was performed using TB Green^®^ Premix Ex Taq^TM^ II reagent (Takara, Dalian, China) on a Bio-Rad CFX96 Real-Time PCR Detection System (Bio-Rad, Hercules, CA, USA). Primers were designed using the NCBI Primer-BLAST online tool (Primer3 v.2.5.0 and BLAST; NCBI, Bethesda, MD, USA; accessed on 28 January 2026). Before formal analysis, the specificity of each primer pair was confirmed by semi-quantitative PCR and melting curve analysis, and primer amplification efficiency was evaluated using serially diluted pooled cDNA. Only primer pairs passing specificity verification and meeting acceptable amplification efficiency standards were applied for relative gene quantification. The primer sequences are listed in Table 2, and *β-actin* was used as the internal reference gene. The PCR conditions were 95 °C for 30 s, followed by 40 cycles of 95 °C for 5 s and 60 °C for 10 s. Relative gene expression levels were calculated using the 2^−ΔΔCt^ method [39]. The FM group was used as the calibrator.

### 2.9. Gut Microbiota Analysis

Gut microbiota analysis was performed by Majorbio Bio-Pharm Technology Co., Ltd. (Shanghai, China). Total genomic DNA extracted from intestinal content samples was used as the template for PCR amplification of the V3–V4 hypervariable region of the bacterial 16S rRNA gene. The primers used were 338F (5′-ACTCCTACGGGAGGCAGCAG-3′) and 806R (5′-GGACTACHVGGGTWTCTAAT-3′) [40]. The purified PCR amplicons were subjected to paired-end sequencing on an Illumina MiSeq platform (Illumina, San Diego, CA, USA). Raw reads were quality-filtered using fastp (version 0.19.6) and merged using FLASH (version 1.2.7) [41,42]. High-quality sequences were clustered and classified into operational taxonomic units (OTUs) using UPARSE (version 7.1) [43]. Bioinformatic analysis was performed using the Majorbio Cloud Platform (https://cloud.majorbio.com (accessed on 20 March 2026)).

### 2.10. Statistical Analysis

The tank was considered the experimental unit for statistical analysis. Unless otherwise stated, all data are presented as the mean ± standard error of the mean (SEM) (n = 4). Statistical analyses were performed using SPSS 26.0 software (IBM, Armonk, NY, USA). Differences among dietary treatments were analyzed by one-way analysis of variance (ANOVA), followed by Tukey’s multiple range test. Significant differences in gut microbiota among treatments were analyzed using the Kruskal–Wallis test with the tool provided by the Majorbio Cloud Platform (https://cloud.majorbio.com (accessed on 20 March 2026)). Differences were considered statistically significant at *p* < 0.05.

## 3. Results

### 3.1. Growth Performance, Feed Utilization and Body Composition

Growth performance, somatic indices, whole body composition and dorsal muscle composition of largemouth bass are shown in Table 3. Compared with the FM control group, the DCSM treatment resulted in statistically significant reductions in FBW, WGR, and SGR (*p* < 0.05). For FBW and SGR, the ECSM and FCSM groups showed intermediate values and did not differ significantly from either the FM or DCSM group (*p* > 0.05). Specifically, the WGR for the ECSM group was significantly lower than that of the FM group (*p* < 0.05), yet the WGR of the FCSM group did not differ significantly from the FM group (*p* > 0.05). Regarding feed efficiency, the DCSM group yielded a significantly higher FCR and a significantly lower PER in comparison to the FM group (*p* < 0.05). However, FCR and PER in the ECSM and FCSM groups were not significantly different from those in either the FM or DCSM group (*p* > 0.05). Furthermore, IBW, SR, FI, FR, HSI, and VSI, as well as whole-body and dorsal muscle proximate compositions, remained not significantly different across all experimental diets (*p* > 0.05).

### 3.2. Serum Biochemical Parameters

Table 4 displays the serum biochemical profiles of the fish fed the various experimental diets. Dietary treatments did not significantly alter the concentrations of TP, ALB, ALT, TG, or GLU (*p* > 0.05). However, the AST activity was significantly higher in the DCSM group than in the FM, ECSM, and FCSM groups (*p* < 0.05), with no significant difference detected among the latter three (*p* > 0.05). In terms of TC, both the DCSM and ECSM groups exhibited significantly higher levels than the FM group (*p* < 0.05). The TC content in the FCSM group showed an intermediate value and was not statistically distinct from any other treatment (*p* > 0.05). Furthermore, the BUN levels were significantly elevated in fish fed the DCSM diet relative to those fed the FM and FCSM diets (*p* < 0.05). The ECSM group presented intermediate BUN levels, with no significant difference when compared with the other experimental groups (*p* > 0.05).

### 3.3. Antioxidant Capacity in Serum, Liver, and Intestine

Antioxidant parameters in the serum, liver, and intestine of largemouth bass fed the experimental diets are shown in Table 5. For the serum samples, SOD, CAT, T-AOC, and MDA levels did not differ significantly among the four dietary groups (*p* > 0.05). However, compared with the DCSM group, the serum GSH-Px activity in the FCSM group was significantly higher (*p* < 0.05), while the groups fed the FM and ECSM diets showed intermediate values (*p* > 0.05). In liver tissue, the activities of SOD and CAT had no significant differences among all treatment groups (*p* > 0.05). In contrast, the hepatic GSH-Px activity in the DCSM-fed fish was significantly lower compared with that in the FM, ECSM, and FCSM groups (*p* < 0.05). The FCSM group also demonstrated significantly higher hepatic T-AOC levels than the FM and DCSM treatments (*p* < 0.05), while the ECSM values remained intermediate. In addition, hepatic MDA content was significantly lower in the FCSM group than in the FM, DCSM, and ECSM groups (*p* < 0.05). In the intestine, SOD and CAT activities were not significantly different among dietary treatments (*p* > 0.05). However, the FCSM treatment significantly increased intestinal GSH-Px activity compared with the FM, DCSM, and ECSM groups (*p* < 0.05) and T-AOC values compared with the FM and DCSM groups (*p* < 0.05). Furthermore, intestinal MDA content was significantly reduced in both the ECSM and FCSM groups compared to the FM and DCSM diets (*p* < 0.05).

### 3.4. Digestive and Intestinal Absorptive Function-Related Enzyme Activities

Table 6 summarizes the data regarding the digestive enzymes and functional enzymatic markers in the stomach and intestine of the tested fish. No significant differences were observed among dietary treatments in gastric pepsin, lipase, or amylase activities (*p* > 0.05). In the intestine, fish fed the FCSM diet showed significantly higher trypsin activity than those fed the FM and DCSM diets (*p* < 0.05), with the ECSM group exhibiting an intermediate level and not differing significantly from the other groups (*p* > 0.05). Intestinal activities of both lipase and amylase were unaffected by the dietary treatments (*p* > 0.05). When evaluating markers of intestinal function, the FCSM group displayed a significantly higher Na^+^/K^+^-ATPase activity than both the FM and DCSM groups (*p* < 0.05), whereas the activity of the ECSM group was intermediate. Moreover, the FCSM diet promoted a significant increase in intestinal γ-GT activity compared with the DCSM diet (*p* < 0.05), whereas the FM and ECSM groups showed intermediate values and did not differ significantly from either the DCSM or FCSM group (*p* > 0.05). However, AKP and CK activities in the intestine did not remain significantly different across all experimental diets (*p* > 0.05).

### 3.5. Intestinal Gene Expression

Intestinal gene expressions are shown in Table 7. Fish fed the DCSM diet showed significantly higher *il1b* and *tnfa* expression and lower *il10* expression than those fed the FM diet (*p* < 0.05). Compared with DCSM, FCSM significantly decreased *il1b* and *tnfa* expression and increased *il10* expression (*p* < 0.05), whereas the ECSM group showed significantly higher *il10* expression than the DCSM group and significantly higher *il1b* expression than the FM and FCSM groups; *tnfa* expression in the ECSM group was intermediate and did not differ significantly from the other groups. The relative expression of other examined genes, including *il8*, *rela*, *zo-1*, *occludin*, *mucin-2*, *tgfb1a*, and *cldn1*, did not differ significantly across the groups (*p* > 0.05).

### 3.6. Gut Microbiota

**Figure 1:**
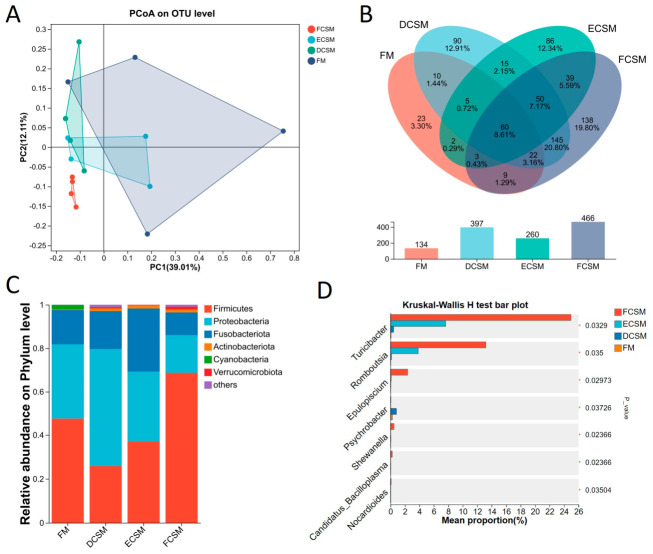
**Gut microbiota analysis of largemouth bass fed different experimental diets.** (**A**) PCoA analysis at the OTU level. (**B**) Venn diagram analysis at the OTU level. (**C**) Histogram of relative abundance at the phylum level. (**D**) Significant difference analysis of gut microbiota abundance at the genus level between multiple groups.

Dietary treatments significantly affected the gut microbiota of largemouth bass (Table 8; Figure 1). In terms of alpha diversity, the FCSM group demonstrated significantly higher Sobs, ACE, Chao1, and Shannon indices compared to the FM control group (*p* < 0.05). Meanwhile, the indices for the DCSM and ECSM groups fell in the intermediate range, with no significant difference from either the FM or FCSM treatment (*p* > 0.05; Table 8). No significant difference was detected in the Simpson index among treatments (*p* > 0.05), and the coverage values in all groups were above 0.999, indicating adequate sequencing depth (Table 8). Principal coordinate analysis (PCoA) demonstrated diet-associated differences in the gut microbiota composition. In particular, the FCSM group formed a distinct cluster and was clearly separated from the other groups (Figure 1A). A Venn diagram illustrated a core microbiome comprising 60 OTUs shared across the four groups. The FCSM treatment showed the greatest microbial richness, harboring 466 total OTUs and 138 unique OTUs (Figure 1B). Taxonomic profiling at the phylum level identified Firmicutes, Proteobacteria, and Fusobacteriota as the predominant bacterial groups across all treatments. The proportion of Firmicutes was higher in the FCSM group, whereas Proteobacteria prevailed in the DCSM and ECSM groups (Figure 1C). At the genus level, multi-species bar plots demonstrated that *Turicibacter* and *Romboutsia* populations were statistically enriched in the FCSM and ECSM groups compared with the DCSM and FM diets, peaking in the FCSM group. Moreover, the FCSM group also showed significantly higher relative abundances of *Epulopiscium*, *Shewanella*, *Candidatus_Bacilloplasma*, and *Nocardioides* than the other groups (*p* < 0.05; Figure 1D). Conversely, *Psychrobacter* was significantly less abundant in the FCSM and ECSM groups than in the DCSM and FM groups (*p* < 0.05; Figure 1D).

## 4. Discussion

The present study showed that the nutritional value of crayfish shell meal for juvenile largemouth bass was strongly influenced by the processing method. Direct inclusion of DCSM impaired growth and feed utilization, whereas FCSM maintained growth and feed utilization indices at levels not significantly different from the FM group and showed more favorable antioxidant, inflammatory, intestinal enzyme, and microbiota responses than DCSM, while ECSM showed partial improvement in several physiological indices. These processing-dependent responses may be mainly associated with changes in nutrient accessibility, lipid oxidation, antioxidant status, intestinal function, and gut microbiota caused by the different processing methods.

Fish fed dried crayfish shell meal exhibited suppressed growth performance and an increased feed conversion ratio, likely because of three related mechanisms. First, the compact chitin–protein–mineral matrix in these by-products acts as a physical barrier that limits the access of digestive enzymes to nutrients, thereby reducing protein digestibility [5]. In addition, chitin is considered a limiting factor in aquafeeds. It can accelerate the passage rate of digesta through the digestive tract, shorten the contact time between nutrients and digestive enzymes, interfere with protease activity, bind lipids, and reduce fat absorption [44]. This interpretation is consistent with previous studies on decapod processing waste, where direct addition of high dietary levels of shrimp shell meal to fish diets significantly reduced growth performance and feed efficiency [45,46,47]. For example, in large yellow croaker (*Larimichthys croceus*), 12% dietary shrimp shell meal did not negatively affect growth performance or feed utilization, whereas 24% dietary shrimp shell meal significantly reduced final body weight, SGR, survival rate, and PER, and increased FCR [47]. These findings indicate that the adverse effects of shrimp shell meal are related to inclusion level and may also depend on species-specific tolerance. Second, beyond these physical barriers, drying and heat treatment may further decrease nutrient availability by promoting protein denaturation and strengthening the association between chitin and protein. Crayfish cephalothorax residues contain residual hepatopancreas tissue, which is rich in highly unsaturated fatty acids (HUFAs) [48], making these residues susceptible to lipid oxidation during drying and heat treatment [49,50]. Third, the intake of poorly digestible chitin-rich material and oxidized lipids may induce downstream physiological stress. Consumption of oxidized lipids or heat-damaged feed often leads to hepatic and intestinal oxidative stress and the accumulation of malondialdehyde (MDA) [51,52]. Mechanistically, lipid peroxidation products such as MDA can damage cellular membranes and impair mitochondrial function, thereby disturbing nutrient metabolism and energy utilization [53,54]. This oxidative stress may also increase the physiological cost of antioxidant defense, inflammatory responses, and tissue repair, thereby partly contributing to reduced growth performance and poorer feed efficiency [53]. In the present study, the DCSM group showed a less favorable antioxidant and MDA profile than the FCSM group, which is consistent with the possible involvement of lipid peroxidation and oxidative stress. However, because lipid oxidation products in the processed crayfish shell meals were not directly measured, this mechanism should be further confirmed in future studies.

Enzymatic hydrolysis, by contrast, represents a milder processing alternative for crayfish by-products. Compared with drying, enzymatic treatment offers certain nutritional and functional advantages because of its mild reaction conditions, which can help retain nutrients and promote the release of specific bioactive peptides. However, the nutritional improvement caused by enzymatic hydrolysis should be interpreted according to the comparison object and experimental context. Studies have demonstrated that enzymatic hydrolysis can improve the nutritional and functional properties of crustacean shell by-products by releasing soluble proteins, peptides, carotenoids, and other bioactive compounds [19,55]. Papain-assisted extraction from shrimp shell waste yields carotenoproteins with a protein recovery of approximately 9–10 g/100 g and a carotenoid content of about 100–115 μg/g. These findings support the ingredient-level nutritional and antioxidant potential of enzyme-treated shell materials, but do not directly verify their growth-promoting effects in fish [19]. Hydrolyzed shell meal has also been reported to enhance nutrient profiles and improve the antioxidant capacity of zebrafish, as dietary supplementation with *Procambarus clarkii* shell protein hydrolysates at 400–1600 mg/kg modulated growth-related indices and enhanced muscle antioxidant capacity [55]. In the present study, the ECSM group exhibited lower intestinal MDA content than the DCSM group, indicating that enzymatic hydrolysis may attenuate intestinal lipid peroxidation associated with dried crayfish shell meal. Nevertheless, the growth performance of fish fed ECSM remained lower than that of the FM group, suggesting that although enzymatic hydrolysis may confer partial physiological benefits, it is insufficient to fully counteract the nutritional limitations imposed by the chitin–protein–mineral matrix of crayfish shell meal.

In contrast, fermented crayfish shell meal resulted in growth performance comparable to that of the control group. The multi-strain fermentation system employed in the present study (*Bacillus subtilis*, *Lactobacillus plantarum*, and *Saccharomyces cerevisiae*) may produce organic acids to demineralize the chitin matrix and secrete exogenous enzymes such as chitinase and protease to facilitate the release and hydrolysis of sequestered proteins [16,22,56,57,58]. Furthermore, the microbial fermentation of crustacean by-products yields hydrolysates rich in carotenoids, peptides, and other bioactive antioxidants with strong free-radical scavenging capabilities [59,60]. Because fermentation occurs under relatively mild, microaerobic, or anaerobic conditions, it may reduce severe thermal damage to the oxidation-prone lipids in the hepatopancreas. These factors likely explain why the partial replacement of fish meal with FCSM did not significantly impair growth and was associated with improved antioxidant indices. Nevertheless, these mechanisms should be interpreted as plausible explanations rather than confirmed causal pathways, as several key intermediate processes were not directly measured in the present study.

Differences in nutrient availability directly impacted the metabolic and absorptive capacities of the fish. The DCSM group showed decreased protein efficiency and elevated blood urea nitrogen, which are indicators of amino acid imbalance [5]. Fermentation treatment alleviated these metabolic disturbances. In the FCSM group, the activities of intestinal trypsin, Na^+^/K^+^-ATPase, and γ-GT were significantly upregulated, which may be attributed to the stimulation of intestinal epithelial cell function by bioactive substances released by microorganisms [61]. Physiologically, trypsin is a key intestinal protease involved in protein hydrolysis, whereas Na^+^/K^+^-ATPase maintains the electrochemical gradient required for epithelial nutrient transport [62,63]. In addition, γ-GT participates in the γ-glutamyl cycle and glutathione metabolism, which contributes to amino acid handling and epithelial redox homeostasis [64]. Their upregulation in the FCSM group therefore suggests enhanced intestinal digestive and absorptive function, partly explaining why growth and feed utilization indices were maintained at levels not significantly different from those of the FM group, together with the more favorable serum and intestinal responses observed in fish fed FCSM.

Regarding immunity, the intestinal mucosal barrier appeared to be highly sensitive to the processing method. Diets containing dried crayfish shell meal induced pro-inflammatory responses, characterized by the upregulation of *il1b* and *tnfa*. This inflammatory response may be associated with mechanical irritation from undigested sharp chitin fragments, combined with the chemical toxicity of oxidized lipids [65,66]. Fermentation mitigated these adverse effects. The resulting immunomodulation is linked to the abundance of bioactive substances in the fermentation products and the favorable modulation of the gut microbiota structure [32,59,60].

The gut microbiota of carnivorous fish exhibits high plasticity [32]. At the phylum level, the FCSM group showed a decrease in Proteobacteria, which are often enriched under dietary stress or intestinal dysbiosis, and an increase in Firmicutes, indicating a more favorable microbial profile [67,68]. This microbial pattern is generally associated with improved gut health and has been reported in aquatic animals fed yeast- or *Lactobacillus*-fermented ingredients [32]. At the genus level, FCSM significantly enriched *Turicibacter*, *Romboutsia*, and *Epulopiscium*. *Turicibacter* is a potentially beneficial genus involved in carbohydrate and lipid metabolism as well as bile acid regulation. Meanwhile, both *Turicibacter* and *Romboutsia* are reported to produce short-chain fatty acids (SCFAs) in the intestine, thereby supporting intestinal barrier function, host metabolism, and immune regulation [69,70]. *Epulopiscium* species are symbiotic bacteria that secrete glycoside hydrolases, thereby enhancing host digestive capacity [71]. Therefore, the enrichment of these potentially beneficial taxa may have contributed to a more favorable intestinal environment, which was consistent with the antioxidant, inflammatory, intestinal enzyme, and gut microbial responses observed in the FCSM group. However, because microbial metabolites such as SCFAs were not measured, the functional implications of these microbial shifts should be further confirmed by metabolomic or metagenomic analyses.

From a practical feed-manufacturing perspective, these findings suggest that crayfish processing by-products should be appropriately treated before being used as feed ingredients for carnivorous fish. Drying is simple and low-cost and is commonly used for preservation. In some commercial processing practices, drying or sanitation treatments may involve temperatures above 100 °C [49], which could aggravate protein denaturation and lipid oxidation in crustacean by-products [18]. In the present study, the poorer growth performance, feed utilization, antioxidant status, and more pronounced inflammatory responses observed in the DCSM group indicate that drying alone may not be sufficient to improve the feeding value of crayfish shell meal. In contrast, fermentation may be a more suitable pretreatment strategy because FCSM maintained growth and feed utilization indices at levels not significantly different from those of the FM group and produced more favorable antioxidant, intestinal enzyme, inflammatory, and gut microbiota responses. Therefore, fermented crayfish shell meal may provide a potential route for partially reducing fish meal use and improving the high-value utilization of crayfish processing waste. For practical feed production, further attention should be paid to processing cost, storage stability, and pellet quality before large-scale application.

Notably, this study still has several limitations. Only one inclusion level of crayfish shell meal and a single feeding duration were tested. In addition, although the diets were formulated to be isonitrogenous and isoenergetic, the Ca:P ratios differed among treatments because of the high mineral content of crayfish shell meal. This dietary mineral variation may have influenced phosphorus availability, mineral utilization, nutrient metabolism, and growth performance, and should therefore be considered a potential nutritional confounding factor when interpreting the present results [72,73]. Moreover, digestibility, ingredient oxidation, histological evaluation, and key functional metabolites were not measured, limiting mechanistic interpretation. Future research should include dose–response experiments and long-term feeding trials, with better control of dietary Ca:P ratio and available phosphorus, together with direct digestibility assays, ingredient oxidation assessment, histological analysis, and metagenomic and metabolomic analyses. These studies are needed to optimize the inclusion level of FCSM, evaluate its long-term effects on fish health, product quality, and economic viability, and clarify the mechanisms by which fermentation improves the utilization of crayfish shell meal.

In summary, dried crayfish shell meal showed limited feeding value for largemouth bass, which may be associated with low nutrient accessibility and heat-related lipid oxidation risk; together, these factors may trigger metabolic stress, lipid peroxidation, and gut dysbiosis. Targeted enzymatic hydrolysis can hydrolyze surface-exposed proteins and partially alleviate physiological stress, but it cannot fully restore growth performance because it fails to disrupt the chitin–protein–mineral matrix. Conversely, multi-strain fermentation may help weaken physical barriers, reduce lipid oxidation risk, and provide potentially beneficial microbial metabolites or substrates. By transforming poorly digestible, oxidation-prone by-products into ingredients with potentially improved digestibility, antioxidant potential, and microbiota-modulating capacity, fermentation represents an effective strategy for the high-value utilization of crayfish processing waste in aquafeeds. However, its practical application should be further optimized through inclusion-level evaluation and long-term feeding validation.

## 5. Conclusions

In conclusion, the present study showed that the processing method markedly affected the nutritional value of crayfish shell meal for largemouth bass. DCSM negatively affected growth and physiological health, whereas enzymatic hydrolysis and, particularly, fermentation alleviated these adverse effects. Rather than serving only as a preservation step, fermentation was an effective pretreatment strategy for transforming crayfish shell meal into a more suitable functional feed ingredient for carnivorous fish. These findings suggest that aquafeed manufacturers may consider fermentation as a preferred processing approach for crustacean by-products before their inclusion in largemouth bass diets, although the optimal inclusion level and long-term effects require further evaluation. In addition, the use of fermented crayfish shell meal may promote the high-value utilization of aquaculture processing waste, reduce disposal pressure, and support the development of more sustainable aquafeeds.

## Figures and Tables

**Table 1:** Feed ingredient composition and nutritional levels (dry matter basis).

Ingredients	Groups			
	FM	DCSM	ECSM	FCSM
Fish meal	45	39.6	39.6	39.6
Dried crayfish shell meal ^1^	0	4.5	0	0
Enzymatically hydrolyzed crayfish shell meal ^2^	0	0	4.5	0
Fermented crayfish shell meal ^3^	0	0	0	4.5
Soybean meal	22	24	24	24
Corn gluten meal	10	10	10	10
Wheat flour	6	5	5	5
Pregelatinized starch	2	2	2	2
Fish oil	5.5	5.5	5.5	5.5
Soybean oil	1.5	1.5	1.5	1.5
Soy lecithin	1.5	1.5	1.5	1.5
Monocalcium phosphate	0.8	0.4	0.4	0.4
Vitamin premix ^4^	1.75	1.75	1.75	1.75
Mineral premix ^5^	1.75	1.75	1.75	1.75
Vitamin C phosphate	0.1	0.1	0.1	0.1
Choline chloride (50%)	0.3	0.3	0.3	0.3
Ethoxyquin	0.02	0.02	0.02	0.02
L-lysine (98%)	0.55	0.55	0.55	0.55
Dl-methionine (99%)	0.4	0.4	0.4	0.4
Taurine	0.5	0.5	0.5	0.5
Zeolite powder	0.33	0.63	0.63	0.63
Total	100	100	100	100
Proximate composition (dry matter basis, %)				
Crude protein (%)	47.23	46.52	46.63	46.78
Crude lipid (%)	12.51	12.32	12.29	12.24
Ash (%)	9.52	10.21	10.07	10.14
Calcium (%)	1.52	1.78	1.69	1.57
Total phosphorus (%)	0.98	0.91	0.90	0.89
Ca:P ratio	1.55:1	1.96:1	1.88:1	1.76:1
Gross energy (MJ/kg)	20.16	19.56	19.71	19.87

^1^ Dried crayfish shell meal: crude protein 38.21%, crude lipid 10.76%, ash 28.53%, and moisture 7.52%. ^2^ Enzymatically hydrolyzed crayfish shell meal: crude protein 38.58%, crude lipid 10.26%, ash 27.69%, and moisture 8.64%. ^3^ Fermented crayfish shell meal: crude protein 39.17%, crude lipid 9.87%, ash 28.43%, and moisture 8.49%. ^4^ Vitamin premix provided the following per kg of diet: vitamin A, 8000 IU; vitamin D3, 1500 IU; vitamin E, 50 IU; vitamin K3, 8 mg; thiamin, 4 mg; riboflavin, 6 mg; pyridoxine, 5 mg; vitamin B12, 0.02 mg; niacin, 40 mg; calcium pantothenate, 20 mg; folic acid, 1.5 mg; biotin, 0.15 mg; inositol, 80 mg; and vitamin C (as L-ascorbyl-2-polyphosphate), 60 mg. Wheat bran was used as the carrier. ^5^ Mineral premix provided the following per kg of diet: Fe (from ferrous sulfate), 20 mg; Cu (from copper sulfate), 1.0 mg; Zn (from zinc sulfate), 40 mg; Mn (from manganese sulfate), 8 mg; I (from potassium iodate), 0.4 mg; Se (from sodium selenite), 0.08 mg; and Co (from cobalt sulfate), 0.2 mg. Zeolite powder was used as the carrier.

**Table 2:** Primers used in this study.

Gene	Primers (5′-3′)	Accession No.	Primer Efficiency (%)
*tgfb1a*	TTGCCAAGGTGTTGCACAAG	XM_038693206.1	98.50%
	CCCCCACACTTTCCCGTATG		
*il10*	AAATCCCAGAGCGGCCTATG	XM_038696252.1	96.20%
	AGACATCACTTCAGCAGGCTC		
*il1b*	CATGGAAGGCGGTAAAGCTG	XM_038733429.1	99.80%
	CCATTTGGGGCAACTGAGAG		
*il8*	CACTCTGCATGCTTCTCCCA	XM_038704089.1	98.10%
	AATTCGGTGAGCAGGACGTT		
*tnfa*	TTGCTTGGTGCTGGAATGGA	XM_038710731.1	97.00%
	GATTCGGCTCAGCGTGTAGT		
*rela*	CAACTCTAAGACCCACCCCG	XM_038730801.1	97.10%
	GCAGTCTTTCCCCACCAGTT		
*zo-1*	GCGTCTATGACCCCTGATGG	XM_038701018.1	100.50%
	CCTGCTGGTCCGTCCTTAAA		
*occludin*	CCAGGGCTCCTCCAGTATGA	XM_038715418.1	99.20%
	CAAGGCGATGACAACCAGGA		
*cldn1*	CCTGTATGGCAGAACAACCG	XM_038718401.1	96.30%
	ACAAACAGGGCACTTCCGAAT		
*mucin-2*	CCACCTCCAGCTCTATCCCT	XM_038705361.1	97.70%
	TGCCAGCACTAATGGTCGTT		
*β-actin*	TGAAACCGGTTCCCTTAAAGC	XM_038695351.1	100.80%
	TTCTGGCCCATACCCACCAT		

**Table 3:** Growth performance, feed utilization, somatic indices, and proximate composition of largemouth bass fed the experimental diets.

Items	Groups			
	FM	DCSM	ECSM	FCSM
Growth performance				
IBW (g)	5.70 ± 0.03	5.67 ± 0.03	5.74 ± 0.03	5.68 ± 0.03
FBW (g)	40.12 ± 1.04 ^b^	33.54 ± 1.27 ^a^	35.27 ± 1.33 ^ab^	37.48 ± 1.04 ^ab^
WGR (%)	604.31 ± 20.52 ^b^	491.69 ± 21.30 ^a^	514.78 ± 21.60 ^a^	564.29 ± 18.23 ^ab^
SGR (%/d)	3.48 ± 0.05 ^b^	3.17 ± 0.06 ^a^	3.24 ± 0.06 ^ab^	3.38 ± 0.05 ^ab^
FCR (g/g)	1.05 ± 0.02 ^a^	1.18 ± 0.02 ^b^	1.13 ± 0.03 ^ab^	1.08 ± 0.02 ^ab^
FI (g/fish/d)	0.65 ± 0.03	0.59 ± 0.03	0.59 ± 0.02	0.62 ± 0.03
FR (%BW/d)	2.81 ± 0.07	2.99 ± 0.08	2.90 ± 0.05	2.85 ± 0.07
PER (g/g)	2.02 ± 0.03 ^b^	1.83 ± 0.03 ^a^	1.90 ± 0.05 ^ab^	1.98 ± 0.04 ^ab^
SR (%)	100.00 ± 0.00	100.00 ± 0.00	100.00 ± 0.00	100.00 ± 0.00
Somatic indices				
HSI (%)	2.63 ± 0.07	2.37 ± 0.09	2.46 ± 0.06	2.52 ± 0.06
VSI (%)	8.48 ± 0.13	8.31 ± 0.10	8.40 ± 0.09	8.51 ± 0.12
Whole body composition				
Moisture (%)	72.33 ± 0.09	72.81 ± 0.14	72.56 ± 0.18	72.46 ± 0.12
Crude protein (%)	17.04 ± 0.13	16.82 ± 0.11	16.68 ± 0.10	16.95 ± 0.10
Crude lipid (%)	4.92 ± 0.11	5.03 ± 0.12	5.34 ± 0.12	5.23 ± 0.06
Ash (%)	3.89 ± 0.06	4.07 ± 0.05	4.02 ± 0.02	3.97 ± 0.03
Dorsal muscle composition				
Moisture (%)	77.25 ± 0.10	77.11 ± 0.14	77.44 ± 0.11	77.19 ± 0.12
Crude protein (%)	19.68 ± 0.10	19.42 ± 0.12	19.34 ± 0.07	19.59 ± 0.07
Crude lipid (%)	1.42 ± 0.07	1.54 ± 0.08	1.66 ± 0.07	1.53 ± 0.04
Ash (%)	1.21 ± 0.02	1.28 ± 0.03	1.26 ± 0.02	1.25 ± 0.02

Data are presented as mean ± SEM (n = 4). Within the same row, values with superscript letters that share no common letter are significantly different (*p* < 0.05), whereas values sharing at least one common superscript letter are not significantly different (*p* > 0.05). Rows without superscript letters indicate no significant differences among treatments (*p* > 0.05). IBW, initial body weight; FBW, final body weight; WGR, weight gain rate; SGR, specific growth rate; FCR, feed conversion ratio; FI, feed intake; FR, feeding rate; PER, protein efficiency ratio; SR, survival rate; HSI, hepatosomatic index; VSI, viscerosomatic index.

**Table 4:** Serum biochemical parameters of largemouth bass fed the experimental diets.

Items	Groups			
	FM	DCSM	ECSM	FCSM
TP (g/L)	43.51 ± 0.52	41.62 ± 1.21	42.33 ± 0.66	42.42 ± 1.04
ALB (g/L)	8.07 ± 0.19	7.81 ± 0.09	7.96 ± 0.15	8.06 ± 0.12
ALT (U/L)	2.99 ± 0.08	3.02 ± 0.21	2.79 ± 0.10	2.91 ± 0.17
AST (U/L)	18.61 ± 1.69 ^a^	24.11 ± 0.77 ^b^	19.20 ± 1.22 ^a^	17.30 ± 0.70 ^a^
TG (mmol/L)	2.04 ± 0.15	2.12 ± 0.05	1.87 ± 0.09	1.98 ± 0.12
TC (mmol/L)	6.70 ± 0.09 ^a^	7.27 ± 0.15 ^b^	7.53 ± 0.11 ^b^	7.11 ± 0.10 ^ab^
GLU (mmol/L)	8.61 ± 0.16	8.85 ± 0.50	8.80 ± 0.27	8.55 ± 0.40
BUN (mmol/L)	1.41 ± 0.03 ^a^	1.79 ± 0.13 ^b^	1.58 ± 0.07 ^ab^	1.47 ± 0.09 ^a^

Data are presented as mean ± SEM (n = 4). Within the same row, values with superscript letters that share no common letter are significantly different (*p* < 0.05), whereas values sharing at least one common superscript letter are not significantly different (*p* > 0.05). Rows without superscript letters indicate no significant differences among treatments (*p* > 0.05). TP, total protein; ALB, albumin; ALT, alanine aminotransferase; AST, aspartate aminotransferase; TG, triglyceride; TC, total cholesterol; GLU, glucose; BUN, blood urea nitrogen.

**Table 5:** Antioxidant parameters in serum, liver and intestine of largemouth bass fed different experimental diets.

Items	Groups			
	FM	DCSM	ECSM	FCSM
Serum				
SOD (U/mL)	26.46 ± 1.57	25.71 ± 0.95	27.73 ± 0.62	29.82 ± 1.25
CAT (U/mL)	2.33 ± 0.10	2.38 ± 0.05	2.54 ± 0.22	2.63 ± 0.33
GSH-Px (U/mL)	29.49 ± 1.14 ^ab^	24.84 ± 2.67 ^a^	31.95 ± 2.12 ^ab^	34.76 ± 0.96 ^b^
T-AOC (U/mL)	1.18 ± 0.09	1.12 ± 0.13	1.32 ± 0.10	1.55 ± 0.10
MDA (nmol/mL)	4.73 ± 0.31	4.84 ± 0.41	4.43 ± 0.24	3.87 ± 0.16
Liver				
SOD (U/mg prot)	46.63 ± 5.12	45.53 ± 2.09	57.10 ± 2.85	55.40 ± 3.67
CAT (U/mg prot)	31.24 ± 0.52	32.55 ± 1.99	36.03 ± 2.26	37.65 ± 0.38
GSH-Px (U/mg prot)	55.21 ± 4.56 ^b^	37.78 ± 2.35 ^a^	49.78 ± 1.08 ^b^	60.30 ± 2.73 ^b^
T-AOC (U/mg prot)	0.56 ± 0.08 ^a^	0.54 ± 0.04 ^a^	0.65 ± 0.05 ^ab^	0.87 ± 0.05 ^b^
MDA (nmol/mg prot)	1.97 ± 0.20 ^b^	2.07 ± 0.14 ^b^	1.83 ± 0.10 ^b^	1.22 ± 0.06 ^a^
Intestine				
SOD (U/mg prot)	32.33 ± 3.35	30.27 ± 1.39	37.87 ± 1.93	38.47 ± 2.49
CAT (U/mg prot)	11.01 ± 0.75	12.58 ± 0.74	14.58 ± 1.07	15.08 ± 1.81
GSH-Px (U/mg prot)	38.81 ± 0.85 ^a^	33.62 ± 1.18 ^a^	44.25 ± 2.63 ^a^	63.90 ± 5.91 ^b^
T-AOC (U/mg prot)	0.69 ± 0.05 ^a^	0.63 ± 0.06 ^a^	0.72 ± 0.09 ^ab^	0.91 ± 0.05 ^b^
MDA (nmol/mg prot)	9.18 ± 0.47 ^b^	9.73 ± 0.41 ^b^	6.81 ± 0.40 ^a^	5.78 ± 0.33 ^a^

Data are presented as mean ± SEM (n = 4). Within the same row, values with superscript letters that share no common letter are significantly different (*p* < 0.05), whereas values sharing at least one common superscript letter are not significantly different (*p* > 0.05). Rows without superscript letters indicate no significant differences among treatments (*p* > 0.05). SOD, superoxide dismutase; CAT, catalase; GSH-Px, glutathione peroxidase; T-AOC, total antioxidant capacity; MDA, malondialdehyde.

**Table 6:** Digestive enzyme activities and related enzyme activities in the stomach and intestine of largemouth bass fed different experimental diets.

Items	Groups			
	FM	DCSM	ECSM	FCSM
Stomach				
Pepsin (U/mg prot)	2114.85 ± 115.97	1962.59 ± 87.43	2324.81 ± 78.47	2386.14 ± 191.38
Lipase (U/mg prot)	19.00 ± 1.49	21.82 ± 3.43	19.04 ± 1.08	19.89 ± 0.77
Amylase (U/mg prot)	0.76 ± 0.06	0.62 ± 0.09	0.65 ± 0.05	0.60 ± 0.05
Intestine				
Trypsin (U/mg prot)	1152.95 ± 33.36 ^a^	1134.54 ± 36.93 ^a^	1271.63 ± 26.80 ^ab^	1448.44 ± 64.15 ^b^
Lipase (U/mg prot)	20.24 ± 1.25	22.43 ± 1.26	23.04 ± 2.64	28.93 ± 5.40
Amylase (U/mg prot)	0.87 ± 0.05	0.91 ± 0.06	0.93 ± 0.05	0.95 ± 0.04
Na^+^/K^+^-ATPase (U/mg prot)	2.95 ± 0.20 ^a^	2.66 ± 0.24 ^a^	3.14 ± 0.30 ^ab^	4.47 ± 0.52 ^b^
AKP (U/mg prot)	38.45 ± 1.55	38.16 ± 2.56	40.49 ± 2.82	42.94 ± 1.91
γ-GT (U/mg prot)	15.14 ± 0.53 ^ab^	13.55 ± 0.92 ^a^	16.89 ± 0.91 ^ab^	19.16 ± 1.72 ^b^
CK (U/mg prot)	1.76 ± 0.09	1.67 ± 0.05	1.98 ± 0.15	1.83 ± 0.13

Data are presented as mean ± SEM (n = 4). Within the same row, values with superscript letters that share no common letter are significantly different (*p* < 0.05), whereas values sharing at least one common superscript letter are not significantly different (*p* > 0.05). Rows without superscript letters indicate no significant differences among treatments (*p* > 0.05). γ-GT, γ-glutamyl transferase; AKP, alkaline phosphatase; CK, creatine kinase.

**Table 7:** Relative gene expressions in the intestine of largemouth bass fed different experimental diets.

Genes	Groups			
	FM	DCSM	ECSM	FCSM
*tgfb1a*	1.00 ± 0.08	0.89 ± 0.18	1.13 ± 0.11	0.98 ± 0.10
*il10*	1.00 ± 0.07 ^b^	0.53 ± 0.07 ^a^	1.14 ± 0.15 ^b^	1.35 ± 0.13 ^b^
*il1b*	1.00 ± 0.08 ^a^	2.01 ± 0.20 ^b^	1.68 ± 0.12 ^b^	0.96 ± 0.12 ^a^
*il8*	1.00 ± 0.09	1.41 ± 0.18	1.32 ± 0.13	1.11 ± 0.16
*tnfa*	1.00 ± 0.10 ^a^	2.31 ± 0.43 ^b^	1.44 ± 0.22 ^ab^	1.23 ± 0.08 ^a^
*rela*	1.00 ± 0.07	1.33 ± 0.08	1.06 ± 0.10	1.13 ± 0.08
*zo-1*	1.00 ± 0.07	0.73 ± 0.10	0.87 ± 0.10	1.10 ± 0.09
*occludin*	1.00 ± 0.06	0.85 ± 0.07	1.20 ± 0.06	1.09 ± 0.14
*cldn1*	1.00 ± 0.06	1.07 ± 0.07	0.92 ± 0.12	1.11 ± 0.13
*mucin-2*	1.00 ± 0.09	0.82 ± 0.08	0.87 ± 0.09	1.14 ± 0.13

Data are presented as mean ± SEM (n = 4). Within the same row, values with superscript letters that share no common letter are significantly different (*p* < 0.05), whereas values sharing at least one common superscript letter are not significantly different (*p* > 0.05). Rows without superscript letters indicate no significant differences among treatments (*p* > 0.05).

**Table 8:** Alpha diversity of gut microbiota in largemouth bass fed different experimental diets.

Alpha Diversity	Groups			
	FM	DCSM	ECSM	FCSM
Sobs	54.50 ± 20.13 ^a^	172.25 ± 35.69 ^ab^	97.25 ± 32.45 ^ab^	232.50 ± 38.04 ^b^
Shannon	1.07 ± 0.25 ^a^	1.93 ± 0.24 ^ab^	1.41 ± 0.41 ^ab^	2.35 ± 0.25 ^b^
Simpson	0.52 ± 0.10	0.26 ± 0.05	0.45 ± 0.16	0.18 ± 0.03
ACE	57.49 ± 19.64 ^a^	178.64 ± 37.06 ^ab^	103.89 ± 34.57 ^ab^	242.96 ± 36.61 ^b^
Chao1	56.11 ± 19.88 ^a^	179.97 ± 37.18 ^ab^	103.08 ± 33.44 ^ab^	244.45 ± 36.61 ^b^
Coverage	0.9999 ± 0.0000	0.9998 ± 0.0001	0.9999 ± 0.0001	0.9997 ± 0.0001

Data are presented as mean ± SEM (n = 4). Within the same row, values with superscript letters that share no common letter are significantly different (*p* < 0.05), whereas values sharing at least one common superscript letter are not significantly different (*p* > 0.05). Rows without superscript letters indicate no significant differences among treatments (*p* > 0.05).

## Data Availability

The raw 16S rRNA gene sequencing data generated in this study have been deposited in the Genome Sequence Archive (GSA) of the China National Center for Bioinformation (CNCB) under project accession number PRJCA064542 and GSA accession number CRA043153. Other data supporting the findings of this study are available from the authors upon reasonable request.

## References

[1] Hua K., Cobcroft J.M., Cole A., Condon K., Jerry D.R., Mangott A., Praeger C., Vucko M.J., Zeng C., Zenger K. (2019). The future of aquatic protein: Implications for protein sources in aquaculture diets. One Earth.

[2] Serra V., Pastorelli G., Tedesco D.E.A., Turin L., Guerrini A. (2024). Alternative protein sources in aquafeed: Current scenario and future perspectives. Vet. Anim. Sci..

[3] Nikoo M., Regenstein J.M., Yasemi M. (2023). Protein hydrolysates from fishery processing by-products: Production, characteristics, food applications, and challenges. Foods.

[4] Ramakrishnan V.V., Hossain A., Dave D., Shahidi F. (2024). Salmon processing discards: A potential source of bioactive peptides—A review. Food Prod. Process. Nutr..

[5] Eggink K.M., Gonçalves R., Skov P.V. (2025). Shrimp processing waste in aquaculture feed: Nutritional value, applications, challenges, and prospects. Rev. Aquac..

[6] Tropea A., Potortì A.G., Lo Turco V., Russo E., Vadalà R., Rando R., Di Bella G. (2021). Aquafeed production from fermented fish waste and lemon peel. Fermentation.

[7] Center N.F.T.E., Fisheries C.S.o. (2025). China crayfish industry development report (2025). China Fish..

[8] Nie F.R., Guo L.X., Li M.Y., Xing L.M., Cheng W. (2021). Evaluation of nutritional value of shrimp shell powder and study on technological parameters of astaxanthin flash extraction. Mod. Anim. Husb..

[9] Cheng X.F., Song R., Hong B., Yuan X.P., He Z.G., Li C.W., Xiang J., Liu L. (2020). Analysis of nutritional components of amino acids and fatty acids in Procrustus clarkii crawfish shell meal. China Feed.

[10] Zhou Y., Guo N., Wang Z., Zhao T., Sun J., Mao X. (2021). Evaluation of a clean fermentation-organic acid method for processing shrimp waste from six major cultivated shrimp species in China. J. Clean. Prod..

[11] Yan N., Chen X. (2015). Sustainability: Don’t waste seafood waste. Nature.

[12] Kaya M., Baran T., Mentes A., Asaroglu M., Sezen G., Tozak K.O. (2014). Extraction and characterization of α-chitin and chitosan from six different aquatic invertebrates. Food Biophys..

[13] Jiang Q.X., Xia W.S. (2004). Study on the factors affecting the recovery of astaxanthin and protein from crayfish processing waste by enzymatic method. Sci. Technol. Food Ind..

[14] Zhang L.H., Gai Y.Q., Wang P., Wang Y.Z., Cheng Z.Y., Zhen T.Y., Piao M.Z., Li Y. (2022). Extraction technology of astaxanthin and chitin from crayfish leftovers. Food Res. Dev..

[15] Tibbetts S.M., Milley J.E., Lall S.P. (2006). Apparent protein and energy digestibility of common and alternative feed ingredients by *Atlantic cod*, *Gadus morhua* (Linnaeus, 1758). Aquaculture.

[16] Abun A., Widjastuti T., Haetami K. (2022). The effect of treatment of shrimp waste with three microbial on nutrient content and digestibility of feed in native chicken. World J. Adv. Res. Rev..

[17] Takeungwongtrakul S., Benjakul S., Aran H. (2012). Lipids from cephalothorax and hepatopancreas of Pacific white shrimp (*Litopenaeus vannamei*): Compositions and deterioration as affected by iced storage. Food Chem..

[18] Takeungwongtrakul S., Benjakul S. (2016). Astaxanthin degradation and lipid oxidation of Pacific white shrimp oil: Kinetics study and stability as affected by storage conditions. Int. Aquat. Res..

[19] Pattanaik S.S., Sawant P.B., Xavier K.A.M., Dube K., Srivastava P.P., Dhanabalan V., Chadha N.K. (2020). Characterization of carotenoprotein from different shrimp shell waste for possible use as supplementary nutritive feed ingredient in animal diets. Aquaculture.

[20] Deng J.J., Zhang M.S., Li Z.W., Lu D.L., Mao H.H., Zhu M.J., Li J.Z., Luo X.C. (2020). One-step processing of shrimp shell waste with a chitinase fused to a carbohydrate-binding module. Green Chem..

[21] Dawood M.A.O., Koshio S. (2019). Application of fermentation strategy in aquafeed for sustainable aquaculture. Rev. Aquac..

[22] Ghorbel-Bellaaj O., Manni L., Jellouli K., Hmidet N., Nasri M. (2012). Optimization of protease and chitinase production by *Bacillus cereus* SV1 on shrimp shell waste using statistical experimental design. Biochemical and molecular characterization of the chitinase. Ann. Microbiol..

[23] Verni M., Verardo V., Rizzello C.G. (2019). How fermentation affects the antioxidant properties of cereals and legumes. Foods.

[24] del Valle J.C., Bonadero M.C., Fernández-Gimenez A.V. (2023). *Saccharomyces cerevisiae* as probiotic, prebiotic, synbiotic, postbiotics and parabiotics in aquaculture: An overview. Aquaculture.

[25] Hoseinifar S.H., Sun Y.-Z., Wang A., Zhou Z. (2018). Probiotics as means of diseases control in aquaculture, a review of current knowledge and future perspectives. Front. Microbiol..

[26] Cai Z.-N., Qian X.-Q., Xie S.-Q. (2020). Optimal dietary protein concentrations for largemouth bass (*Micropterus salmoides*) of different sizes (10–500 g). Aquac. Int..

[27] Huang D., Wu Y., Lin Y., Chen J., Karrow N., Ren X., Wang Y. (2017). Dietary protein and lipid requirements for juvenile largemouth bass, *Micropterus salmoides*. J. World Aquac. Soc..

[28] Liu Y., Pu C., Pei Z., Zhang W., Wei Z., Chen H., Huang Y. (2025). Retrospect of fishmeal substitution in largemouth bass (*Micropterus salmoides*): A review. Fish Physiol. Biochem..

[29] Martínez-Álvarez R.M., Morales A.E., Sanz A. (2005). Antioxidant defenses in fish: Biotic and abiotic factors. Rev. Fish Biol. Fish..

[30] Ringø E., Zhou Z., Vecino J.L.G., Wadsworth S., Romero J., Krogdahl Å., Olsen R.E., Dimitroglou A., Foey A., Davies S. (2016). Effect of dietary components on the gut microbiota of aquatic animals. A never-ending story?. Aquac. Nutr..

[31] Xu F.-M., Hou S.-W., Wang G.-X., Gong J.-Y., Zhou L., Huang Y.-H., Huang X.-D., Liu L. (2021). Effects of zymolytic black soldier fly (*Hermetia illucens*) pulp as dietary supplementation in largemouth bass (*Micropterus salmoides*). Aquac. Rep..

[32] Siddik M.A.B., Julien B.B., Islam S.M.M., Francis D.S. (2024). Fermentation in aquafeed processing: Achieving sustainability in feeds for global aquaculture production. Rev. Aquac..

[33] Wang Q., Qi Z., Fu W., Pan M., Ren X., Zhang X., Rao Z. (2024). Research and prospects of enzymatic hydrolysis and microbial fermentation technologies in protein raw materials for aquatic feed. Fermentation.

[34] Mondal K., Kaviraj A., Mukhopadhyay P.K. (2011). Partial replacement of fishmeal by fermented fish-offal meal in the formulation of diet for Indian minor carp Labeo bata. J. Appl. Aquac..

[35] Liu J., Gao Q., Bian C., Ma Q., Wei Y., Liang M., Xu H. (2025). An evaluation of chlorella (*Chlorella pyrenoidosa*) in the feed of juvenile tiger puffer (*Takifugu rubripes*). Fishes.

[36] Horwitz W., Latimer G.W. (2005). Official Methods of Analysis of AOAC International.

[37] (2018). Determination of Calcium in Feeds.

[38] (2018). Determination of Phosphorus in Feeds—Spectrophotometry.

[39] Rao X., Huang X., Zhou Z., Lin X. (2013). An improvement of the 2ˆ(–delta delta CT) method for quantitative real-time polymerase chain reaction data analysis. Biostat. Bioinform. Biomath..

[40] Liu C., Zhao D., Ma W., Guo Y., Wang A., Wang Q., Lee D.-J. (2015). Denitrifying sulfide removal process on high-salinity wastewaters in the presence of *Halomonas* sp.. Appl. Microbiol. Biotechnol..

[41] Chen S., Zhou Y., Chen Y., Gu J. (2018). Fastp: An ultra-fast all-in-one FASTQ preprocessor. Bioinformatics.

[42] Magoč T., Salzberg S.L. (2011). FLASH: Fast length adjustment of short reads to improve genome assemblies. Bioinformatics.

[43] Edgar R.C. (2013). UPARSE: Highly accurate OTU sequences from microbial amplicon reads. Nat. Methods.

[44] Pascon G., Opere Akinyi R., Cardinaletti G., Daniso E., Messina M., Tulli F. (2025). Chitin and its effects when included in aquafeed. Aquac. Int..

[45] Lu C.-H., Ku C.-C. (2013). Effects of shrimp waste meal on growth performance and chitinase activity in juvenile cobia (*Rachycentron canadum*). Aquac. Res..

[46] Hansen A.C., Rosenlund G., Karlsen Ø., Olsen R.E., Hemre G.I. (2013). Marine ash-products influence growth and feed utilization when *Atlantic cod Gadus morhua* L. are fed plant-based diets. J. Appl. Ichthyol..

[47] Yi X., Li J., Xu W., Zhou H., Smith A.A., Zhang W., Mai K. (2015). Shrimp shell meal in diets for large yellow croaker Larimichthys croceus: Effects on growth, body composition, skin coloration and anti-oxidative capacity. Aquaculture.

[48] Qin G., Chen H., Liu F., Chu T., Wang M., Lou B., Qian H., Yao Z. (2022). Changes of lipid composition and fatty acid content in the hepatopancreas and expression of FABP before and after molting in red claw crayfish (*Cherax quadricarinatus*). J. World Aquac. Soc..

[49] Tan H., Huang Q., Yu J., Lu Y., Wei L., Shi L., Yu W., Qiao Y. (2025). Analysis of factors affecting the browning of crayfish hepatopancreas after high-temperature sterilization. LWT.

[50] Fitri N., Chan S.X.Y., Che Lah N.H., Jam F.A., Misnan N.M., Kamal N., Sarian M.N., Mohd Lazaldin M.A., Low C.F., Hamezah H.S. (2022). A comprehensive review on the processing of dried fish and the associated chemical and nutritional changes. Foods.

[51] Zhang D.-G., Zhao T., Hogstrand C., Ye H.-M., Xu X.-J., Luo Z. (2021). Oxidized fish oils increased lipid deposition via oxidative stress-mediated mitochondrial dysfunction and the CREB1-Bcl2-Beclin1 pathway in the liver tissues and hepatocytes of yellow catfish. Food Chem..

[52] Yu H., Ren Y., Wei H., Xing W., Xu G., Li T., Xue M., Luo L. (2022). Dietary oxidized fish oil negatively affected the feed utilization, health status and fillet quality of juvenile Amur sturgeon, *A. schrenckii*. Aquaculture.

[53] Yin P., Xie S., Huo Y., Guo T., Fang H., Zhang Y., Liu Y., Tian L., Niu J. (2019). Effects of dietary oxidized fish oil on growth performance, antioxidant defense system, apoptosis and mitochondrial function of juvenile largemouth bass (*Micropterus salmoides*). Aquaculture.

[54] Gaschler M.M., Stockwell B.R. (2017). Lipid peroxidation in cell death. Biochem. Biophys. Res. Commun..

[55] Li H., Isaac N., He S., Ou X., Xiang J., Tian X., Zhu H., Liu L., Wen L., Chu W. (2022). Dietary supplementation with protein hydrolysates from the shell of red swamp crayfish (*Procambarus clarkii*) affects growth, muscle antioxidant capacity and circadian clock genes expression of zebrafish (*Danio rerio*). Aquac. Rep..

[56] Mathew G.M., Mathew D.C., Sukumaran R.K., Sindhu R., Huang C.-C., Binod P., Sirohi R., Kim S.-H., Pandey A. (2020). Sustainable and eco-friendly strategies for shrimp shell valorization. Environ. Pollut..

[57] Zhang Q., Wang L., Liu S., Li Y. (2021). Establishment of successive co-fermentation by *Bacillus subtilis* and *Acetobacter pasteurianus* for extracting chitin from shrimp shells. Carbohydr. Polym..

[58] Abun T.W., Haetami K. (2016). Effect of time processing at steps of bioprocess shrimp waste by three microbes on protein digestibility and metabolizable energy products of native chicken. Agrolife Sci. J..

[59] Cremades O., Ponce E., Corpas R., Gutiérrez J.F., Jover M., Alvarez-Ossorio M.C., Parrado J., Bautista J. (2001). Processing of crawfish (*Procambarus clarkii*) for the preparation of carotenoproteins and chitin. J. Agric. Food Chem..

[60] Ghorbel-Bellaaj O., Younes I., Maalej H., Hajji S., Nasri M. (2012). Chitin extraction from shrimp shell waste using *Bacillus* bacteria. Int. J. Biol. Macromol..

[61] Assan D., Kuebutornye F.K.A., Hlordzi V., Chen H., Mraz J., Mustapha U.F., Abarike E.D. (2022). Effects of probiotics on digestive enzymes of fish (finfish and shellfish); status and prospects: A mini review. Comp. Biochem. Physiol. Part B Biochem. Mol. Biol..

[62] Solovyev M., Kashinskaya E., Gisbert E. (2023). A meta-analysis for assessing the contributions of trypsin and chymotrypsin as the two major endoproteases in protein hydrolysis in fish intestine. Comp. Biochem. Physiol. Part A Mol. Integr. Physiol..

[63] Hedén I., Sundell K., Jönsson E., Sundh H. (2022). The role of environmental salinity on Na+-dependent intestinal amino acid uptake in rainbow trout (*Oncorhynchus mykiss*). Sci. Rep..

[64] Griffith O.W., Bridges R.J., Meister A. (1979). Transport of gamma-glutamyl amino acids: Role of glutathione and gamma-glutamyl transpeptidase. Proc. Natl. Acad. Sci. USA.

[65] Valacchi G., Magnani N., Woodby B., Ferreira S.M., Evelson P. (2020). Particulate matter induces tissue oxinflammation: From mechanism to damage. Antioxid. Redox Signal..

[66] Sottero B., Rossin D., Poli G., Biasi F. (2018). Lipid oxidation products in the pathogenesis of inflammation-related gut diseases. Curr. Med. Chem..

[67] Shin N.-R., Whon T.W., Bae J.-W. (2015). Proteobacteria: Microbial signature of dysbiosis in gut microbiota. Trends Biotechnol..

[68] Stojanov S., Berlec A., Štrukelj B. (2020). The influence of probiotics on the Firmicutes/Bacteroidetes ratio in the treatment of obesity and inflammatory bowel disease. Microorganisms.

[69] Lynch J.B., Gonzalez E.L., Choy K., Faull K.F., Jewell T., Arellano A., Liang J., Yu K.B., Paramo J., Hsiao E.Y. (2023). Gut microbiota *Turicibacter* strains differentially modify bile acids and host lipids. Nat. Commun..

[70] Fan Z., Ke X., Jiang L., Zhang Z., Yi M., Liu Z., Cao J., Lu M., Wang M. (2024). Genomic and biochemical analysis reveals fermented product of a putative novel *Romboutsia* species involves the glycometabolism of tilapia. Aquaculture.

[71] Ngugi D.K., Miyake S., Cahill M., Vinu M., Hackmann T.J., Blom J., Tietbohl M.D., Berumen M.L., Stingl U. (2017). Genomic diversification of giant enteric symbionts reflects host dietary lifestyles. Proc. Natl. Acad. Sci. USA.

[72] Hossain M.A., Yoshimatsu T. (2014). Dietary calcium requirement in fishes. Aquac. Nutr..

[73] Wang P., Li X., Xu Z., Ji D., He M., Dang J., Leng X. (2022). The digestible phosphorus requirement in practical diet for largemouth bass (*Micropterus salmoides*) based on growth and feed utilization. Aquac. Fish..

